# Anders, maar ook minder?

**DOI:** 10.1007/s13629-020-00295-7

**Published:** 2020-08-04

**Authors:** Peter F. A. Mulders

**Affiliations:** Afdeling Urologie, Radboud Universitair Medisch Centrum Nijmegen, Nijmegen, Nederland

Ieder gesprek bevat tegenwoordig in een van de eerste zinnen het woord Corona. Zo ook dit editorial, met een hoofdletter geschreven vanwege de impact van het virus op alles wat we doen. Een tijdschrift heeft daar in eerste instantie minder last van. De vergaderingen van de editorial board gingen al telefonisch en correspondentie on line, Coronaproof. Een veel later effect is dat de hoeveelheid kopij zal afnemen; er worden minder patiënten gezien en veel research ligt relatief stil. Gelukkig komen activiteiten in de urologische kliniek weer op gang, maar dat het anders zal worden, is inmiddels wel duidelijk.

De artikelen in dit nummer gaan over zorg die een tijd stil heeft gelegen; ze werden ook al vóór de Coronacrisis ter review aangeboden. Het artikel van Jacobs en collega’s gaat over PROM/PREM’s bij lithiasis. Een interessant onderzoeksthema over een onderwerp dat we vooral van Davinci-prostatectomieën kennen. Mekke en collega’s geven in het daarop volgende artikel het TURP/blaassteendilemma goed weer, waarna Jansen en collega’s de rol van de testisprothese nog eens onder de aandacht brengen.

Nog een gevolg van Corona: COVID-19 heeft ervoor gezorgd dat we elkaar niet fysiek ontmoeten tijdens het EAU-congres van dit jaar. Als het goed is, gaat dat echter wel in 2022 gebeuren, ook – net zoals nu de bedoeling was – in Amsterdam. Dus we krijgen als Nederlandse urologen nóg een keer de kans om dit event in ons land te hosten, alhoewel het de vraag is of dat weer met 15.000 deelnemers zal zijn. De EAU 2020 is dus online en alle abstract zullen ook langs die weg gereviewd worden volgens het junior/senior EAU-reviewprincipe. Het geeft ongetwijfeld weer een goed overzicht van de laatste ontwikkelingen op ons vakgebied, ontwikkelingen die nog niet door Corona zijn beïnvloed.

Dus, veel leesplezier, daar hebben we meer tijd voor, en blijf Coronavrij (en dat is zes).

